# Standard-Dose Atorvastatin Treatment in Patients With Symptomatic Middle Cerebral Artery Atherosclerotic Stenosis: A Vessel Wall Magnetic Resonance Imaging Study

**DOI:** 10.3389/fneur.2021.693397

**Published:** 2021-12-08

**Authors:** Yejun Wu, Fangbing Li, Yilin Wang, Tianxiang Hu, Honghua Gao

**Affiliations:** ^1^Department of Radiology, Fourth Affiliated Hospital of China Medical University, Shenyang, China; ^2^Department of Neurology, Fourth Affiliated Hospital of China Medical University, Shenyang, China

**Keywords:** vessel wall magnetic resonance imaging, middle cerebral artery, atorvastatin, stand-dose, atherosclerotic stenosis

## Abstract

**Background and Purpose:** Ischemic stroke can be caused by atherosclerotic lesions of the middle cerebral artery (MCA). Some studies have described the effects of statin treatment on carotid artery plaques, but little is known about the effects of statin treatment on MCA plaques. The purpose of this study was to validate the efficacy of standard-dose atorvastatin (20 mg/day) in patients with symptomatic MCA atherosclerotic stenosis (SMAS) in northern China.

**Materials and Methods:** This study is a prospective, single-arm, single-center, 12-month follow-up observational study monitoring imaging, and clinical outcomes of standard-dose atorvastatin treatment among patients with SMAS. The primary outcomes were changes in vessel wall magnetic resonance imaging (VWMRI) and serum lipid profiles before and after (1, 3, 6, and 12 months) statin treatment.

**Results:** A total of 46 patients were recruited for this study, and 24 patients completed the follow-up. During the follow-up period, serum non-high-density lipoprotein cholesterol concentrations gradually decreased in the patients. Fourteen patients (54.33%) had a reversal of MCA plaques and 10 patients (41.67%) had no significant progression of MCA plaques and remained stable at the follow-up endpoint. At the 12 months follow-up time-point, the treatment did not reverse vascular remodeling or change the shape and distribution of plaques. Altered serum low-density lipoprotein cholesterol (LDL-C) concentrations in patients were strongly associated with plaque reversal.

**Conclusion:** Vessel wall magnetic resonance imaging could accurately characterize changes in MCA plaques after lipid-lowering therapy. Standard-dose atorvastatin treatment could stabilize and reverse plaques in northern Chinese patients with SMAS.

## Introduction

Intracranial atherosclerotic disease (ICAD) is one of the major causes of ischemic stroke worldwide, and it is more common in the Chinese population (1). The middle cerebral artery (MCA) is an important branch of the intracranial carotid artery and is most susceptible to atherosclerotic lesions. Intracranial atherosclerotic disease can cause cerebral tissue ischemia, and thus, a variety of neurological symptoms through the following mechanisms: occlusion of blood vessels by thrombi, occlusion of small penetrating arteries, artery-to-artery embolism due to plaque rupture, and inadequate perfusion of brain tissue due to intracranial arterial stenosis (2). Therefore, treatment strategies for ICAD include the following: first, anti-thrombotic drugs (3). Second, percutaneous transluminal angioplasty and stenting (4). Third, the adoption of healthy lifestyle choices and aggressive control of ICAD risk factors, such as low-density lipoprotein cholesterol (LDL-C) control below 70 mg/dl and systolic blood pressure control below 140 mm/Hg (5).

Previous studies have shown that statins can cause plaque stabilization or reversal in carotid arteries by lowering the serum LDL-C and triglyceride concentrations (6, 7) and reducing plaque lipid content (8, 9). Moreover, statins have been shown to slow down the progression of atherosclerosis and reduce the incidence of cerebrovascular events in patients with stroke through their effective lipid-lowering function (10). Several studies have confirmed that high-dose statins stabilize plaques in patients with ICAD (11, 12). However, as the statin dose increases, the risk of adverse effects in patients increases (13, 14). To our current knowledge, there are no detailed reports on whether the stabilization and reversal of atherosclerotic plaques in the brain occur after patients with symptomatic MCA atherosclerotic stenosis (SMAS) are treated with standard doses of statins (atorvastatin, 20 mg/day), and how early these changes occur. Vessel wall magnetic resonance imaging (VWMRI) is currently recognized as the best non-invasive method for evaluating the vascular characteristics of intracranial atherosclerotic lesions. This technique allows for the assessment of the intracranial vascular lumen and the vessel wall (15). Therefore, we designed and conducted this prospective, single-arm, 12-month follow-up observational study using VWMRI to assess the correlation between changes in lipid levels and changes in MCA plaques in patients treated with standard doses of atorvastatin for SMAS. We hypothesized that VWMRI would allow the precise assessment of plaque changes during atorvastatin treatment in patients with SMAS and provide rich information for evaluating atorvastatin drug efficacy.

## Materials and Methods

### Study Design

This study is a 12-month single-center, single-arm, prospective, observational study focused on monitoring changes in imaging and clinical outcomes in patients with SMAS who were taking standard doses of atorvastatin (Lipitor, Pfizer, Inc., USA; 20 mg/day). The study protocol and informed consent were reviewed and approved by the Ethics Committee of our hospital, and the study was registered with the China Clinical Trials Registry. All patients signed an informed consent form.

Patients were recruited from the inpatients of the Department of Neurology of our hospital from March 21 to December 31, 2019. Due to the lack of prior relevant literature, it was impossible to determine the sample size required to observe the changes in the responsible vascular imaging features of patients with SMAS over 12 months of treatment with standard-dose atorvastatin. Considering the study period and available study funding, we planned to enroll 50 patients.

Inclusion criteria: (1) age 20–80 years (2) atorvastatin treatment began upon enrollment in the study and the patients had not previously taken atorvastatin (3) presence of one or more atherosclerotic risk factors (4) confirmed diagnosis of SMAS [diagnosis made by a neurologist according to the diagnostic criteria for intracranial artery stenosis (16)] (5) recent (14 days) ischemic stroke or transient ischemic symptoms (6) not treated with angioplasty for intracranial artery stenosis.

Exclusion criteria: (1) contraindication to MRI (2) severe hepatic or renal dysfunction or malignancy (3) extracranial artery stenosis >50% (4) atrial fibrillation, severe cardiac insufficiency, cardiogenic stroke risk factors (5) incomplete clinical data (6) MCA stenosis due to non-atherosclerotic lesions.

All patients who met the inclusion criteria received 12 months of standard-dose atorvastatin treatment. Clinical information was recorded for all patients. All patients received VWMRI and blood biochemistry analysis to determine baseline levels before treatment, and VWMRI and blood analyses were repeated at months 1, 3, 6, and 12 after treatment. In addition, all patients received anti-platelet therapy, with blood pressure control in patients with hypertension (target blood pressure below 140/90 mm/Hg; hypertension with diabetes, target blood pressure below 130/80 mm/Hg), and active glycemic control in patients with diabetes (fasting blood glucose control below 7 mmol/L and postprandial blood glucose control below 11 mmol/L). Clinical and home blood pressure and glucose monitoring were performed to clarify blood pressure and glucose control during the study period.

### Follow-Up and Evaluation of Clinical Outcomes

The main clinical indicators tested included (1) the vascular characteristics and changes in the MCA by VWMRI before and after atorvastatin treatment. The blood biochemical examination included changes in serum triglycerides, total cholesterol, LDL-C, and high-density lipoprotein cholesterol (HDL-C). Phosphocreatine kinase and liver transaminases were evaluated to determine if they were in the normal or abnormal range and (2) whether clinical cerebrovascular events (including cerebrovascular death, recurrent transient ischemic attack, ischemic stroke) occurred during the study period. (3) Vital signs and neurological-specific physical examination, dietary status questioning, and medication compliance assessments were conducted.

### VWMRI Protocol

Vessel wall magnetic resonance imaging was performed on a 3.0T MRI (GE Discovery MR750; Milwaukee, WI, USA) with an eight-channel head coil, and the scan parameters are shown in Table 1. To localize the artery of interest, a three-dimensional time-of-flight MRA (3D TOF MRA) of the circle of Willis was performed. The 3D TOF-MRA images from each patient were used as the positioning images to scan the 3D T1-weighted imaging (T1WI)/2D T2-weighted imaging (T2WI). The MRI scanning equipment, patient's scanning position, range, and scan parameters were consistent at each follow-up time point.

**Table 1:** Vessel wall imaging protocol.

**Sequences**	**TR (ms)**	**TE (ms)**	**Flip angle**	**Slice thickness (mm)**	**FOV (mm)**	**NEX**	**Locs per slab**	**Matrix**	**Acquisition time**
3D TOF-MRA	23	2.5	20	1.4	220	3	32	320 × 256	4 min 1 s
3D CUBE T1WI	1,140	14	/	1	180	1	160	320 × 228	7 min 5 s
3D CUBE PDWI	2,500	35	/	1	180	1	160	320 × 228	9 min 46 s
2D FSE T2WI	4,000	42	125	2	130	4	16	256 × 224	6 min 8 s

*3D, three dimensional; 2D, two dimensional; PDWI, proton density-weighted imaging; TR, repetition time; TE, echo time; FOV, field of view; NEX, number of excitations; Locs per slab, total number of locations (slices) generated from a slab; min, minute; s, second; CUBE, variable-flip-angle turbo-spin-echo*.

### Definition of Different Features of VWMRI

The Warfarin Aspirin Symptomatic Intracranial Disease criteria are widely used to measure MCA stenosis (stenosis rate, %) = [1 – (diameter of stenosis/diameter of normal) × 100%)] (17). The vessel area and lumen area are measured at the narrowest part of the MCA. Plaque burden is defined as (vessel area – lumen area)/vessel area × 100%.

The ratio between the vascular area at the plaque and the vascular area proximal to the plaque is defined as the remodeling rate (RR). An RR > 1.05 is positive remodeling, an RR <0.95 is negative remodeling, and an RR between 0.95 and 1.05 is defined as no remodeling (18, 19). Plaque surface irregularities can be considered when the plaque surface is discontinuous or poorly displayed. Plaque distribution was divided on the cross-sectional images of the MCA (20) and averaged into four distributions: ventral, dorsal, superior, and inferior quadrants. The quadrant with the most significant proportion of plaque was taken to determine the plaque distribution. The eccentricity index (maximum vessel wall thickness – minimum vessel wall thickness)/maximum vessel wall thickness) was used to evaluate the shape of the plaque, with an eccentricity index ≥0.5 for eccentric vessel wall thickening and otherwise for centripetal (annular) vessel wall thickening (21).

### Image Analysis

First, a radiologist assessed the image quality of the VWMRI. The image quality was then divided into four levels based on the clarity of the vessel wall structure in the analyzed imaging sequence: level 1, non-diagnostic; level 2, not suitable for diagnostic purposes; level 3, adequate for diagnostic purposes; level 4, high quality for diagnostic purposes (22). Images with quality below level 3 were excluded and were not used for statistical analyses. Secondly, two other radiologists separately and independently measured and evaluated the VWMRI images using a blinded method according to the above criteria. The patients' information and follow-up times were not visible to the radiologists during the measurement and evaluation. One of the radiologists re-evaluated the VWMRI images 4 weeks later to assess the inter-observer agreement. All measurements were performed on a GE workstation. The measurements of two radiologists were averaged and used for the final analyses.

Since continuous or diffuse lesions are widespread in MCA stenosis, measurements of diffuse lesions in the MCA were made for the most severe lesions. The lumen is manually outlined on T1WI, the outer wall of the vessel is manually drafted on T2WI, and the GE workstation automatically calculates the lumen and vessel areas.

### Laboratory Tests

The patients were examined between 8:00 to 10:00 a.m. Serum was obtained by centrifugation (6 min at 4,000 rpm), and an automatic biochemical analyzer (ADVIA2400, Siemens, Berlin, Germany) was used to obtain the biochemical measurements. An immune transmission turbidimetric method was used to measure the serum triglycerides (normal: 0.38–2.83 mmol/l), total cholesterol (normal: 3.50–5.18 mmol/l), LDL-C (normal: 2.60–3.40 mmol /L), HDL-C (normal 0.95–1.95 mmol /L) concentration; phosphoric creatine kinase (normal: 32–294 units/l), and liver transaminase activity (glutamic–pyruvic transaminase normal: 7–40 units/l; glutamic-oxaloacetic transaminase normal: 19–35 units/l).

### Statistics

All statistical analyses were performed using a statistical software (MedCalc v19.0.7; MedCalc Software Ltd., Ostend, Belgium). The Shapiro-Wilk test was used to verify whether continuous variables conformed to a normal distribution. Data with a normal distribution were expressed as mean ± SD. Data with a non-normal distribution were expressed as the median and interquartile range (median spacing: 25–75%). For discrete variables, data are expressed as counts and percentages. Intra- and inter-observer agreement were evaluated using the intraclass correlation coefficient (ICC), with good agreement for ICC > 0.80; 0.40 ≤ ICC ≤ 0.80, fair agreement; and poor agreement for ICC < 0.4. Serum values and magnetic resonance measurements at each follow-up time point were compared with baseline time points using a signed rank-sum test or paired *t*-test. Multiple linear regression analysis was further used to explore independent clinical and imaging factors influencing changes in plaque loading and stenosis, and variables with *P* < 0.20 in univariate analysis were considered explanatory variables and evaluated in subsequent multivariate analysis. *P* < 0.05 was considered to be statistically significant for all statistical analyses.

## Results

### Patient Clinical Information

A total of 46 patients were recruited during the study period, and 24 completed the 12-month follow-up (14 missed visits, 1 discontinued intervention due to adverse drug reactions, 1 underwent MCA angioplasty during the follow-up period, 1 had a sudden onset of other illnesses, and 5 were affected by the COVID-19 epidemic). The clinical information for all patients who completed follow-up at baseline time points is shown in Tables 2, 3. The lifestyle changes in patients who completed follow-up included: low-fat diet (23), low-salt diet (15), diabetic diet (9), smoking cessation (7), and alcohol cessation (5). Seven people with a history of hypertension had blood pressure control below 130/80 mm/Hg, four people had blood pressure control below 140/90 mm/Hg, three people had average blood pressure control, with blood pressure fluctuations ranging from 130 to 150/80 to 100 mm/Hg, and one person had poor blood pressure control. All nine people with a history of diabetes mellitus attained glycemic control. The follow-up endpoint National Institute of Health Stroke Scale (NIHSS) score was 0 (0–1), and there were no new abnormal neurological symptoms, ischemic stroke, or recurrence of transient ischemic attack during the follow-up period. All patients who completed the follow-up had good compliance with drug therapy. No adverse drug reactions occurred during the follow-up period, except for one person with abnormal phosphocreatine kinase metabolism.

**Table 2:** Patients' baseline clinical information.

	***N* = 24**
Age (year)	55.26 ± 10.31
Sex	
Male	11 (45.83%)
Female	13 (54.17%)
Body mass index (kg/m^2^)	24.86 ± 0.48
Smoking (ever)	7 (29.17%)
Diabetes	9 (37.50%)
Hypertension	15 (62.50%)
Dyslipidemia	13 (54.16%)
Alcoholism (ever)	5 (20.83%)
NIHSS	1^*^ (0–3)
Clinical events	
Transient ischemic attack	3 (12.50%)
Ischemic stroke	21 (87.50%)
Serum lipoprotein concentration (mmol/L)	
Total cholesterol	3.79 ± 1.02
Triglycerides	1.77 ± 0.97
Low-density lipoprotein cholesterol	2.17 ± 1.00
High-density lipoprotein cholesterol	0.95 ± 0.33
Phosphocreatine kinase and hepatic transaminase activity	
Normal	24 (100.00%)
Abnormal	0 (0.00%)
Anti-platelet drug	
Aspirin (100 mg/day)	19
Clopidogrel (75 mg/day)	5

*NIHSS, National Institutes of Health Stroke Scale*;

* *21 patients with ischemic stroke*.

**Table 3:** Patients' serum lipid concentrations during the follow-up.

**Serum lipids (mmol/L)**	**Baseline**	**1 month**	**3 month**	**6 month**	**12 month**
	**(*N* = 24)**	**(*N* = 24)**	**(*N* = 24)**	**(*N* = 24)**	**(*N* = 24)**
Total cholesterol	3.79 ± 1.20	3.60 ± 0.97	3.50 ± 1.07	3.37 ± 0.97	3.09 ± 0.78
Triglycerides	1.77 ± 0.97	1.66 ± 0.89	1.43 ± 0.62	1.35 ± 0.58	1.23 ± 0.50
Low-density lipoprotein cholesterol	2.17 ± 1.00	2.04 ± 0.73	1.93 ± 0.78	1.89 ± 0.72	1.68 ± 0.59
High-density lipoprotein cholesterol	0.95 ± 0.33	1.00 ± 0.22	1.06 ± 0.42	1.09 ± 0.43	0.96 ± 0.31

### Changes in VWMRI in Patients

A total of 120 images were obtained from patients at all follow-up time points, and the image quality met the criterium of level 3 or higher (level 3: 27, level 4: 93). Data were collected for: stenosis rate [intra- and inter-observer: 0.94 (0.90–0.95), 0.93 (0.87–0.96)], plaque burden [intra- and inter-observer: 0.96 (0.91–0.98), 0.95 (0.93–0.97)], RR [intra- and inter-observer: 0.97 (0.87–0.99),0.95 (0.86–0.97)], and eccentricity index [intra- and inter-observer: 0.97 (0.89–0.99),0.98 (0.90–0.99)]. All data had good intra- and inter-observer agreement.

There were 12 cases of severe MCA stenosis (stenosis rate: 75.35 ± 6.35%), 11 cases of moderate stenosis (stenosis rate: 61.25 ± 5.07%), and 1 case of mild stenosis (stenosis rate: 31.23%) in patients atbaseline at the commencement of the study. Vessel remodeling observed: there were 7 cases of negative remodeling (RR: 0.91 ± 0.02), 6 cases of positive remodeling (RR: 1.11 ± 0.04), and 11 cases of no remodeling (RR:1.01 ± 0.01). Plaque shape and distribution observed: there were 22 cases of eccentric thickening (10 cases of plaque distribution in the ventral wall, 6 cases in the inferior wall, 2 cases in the superior wall, and 4 cases in the dorsal wall), and 2 cases of centripetal thickening (circumferential wall: 2 cases). The mean plaque burden was 81.30 ± 16.38%. Eleven patients (11/24, 45.83%) had rough plaque surfaces.

**Figure 1:**
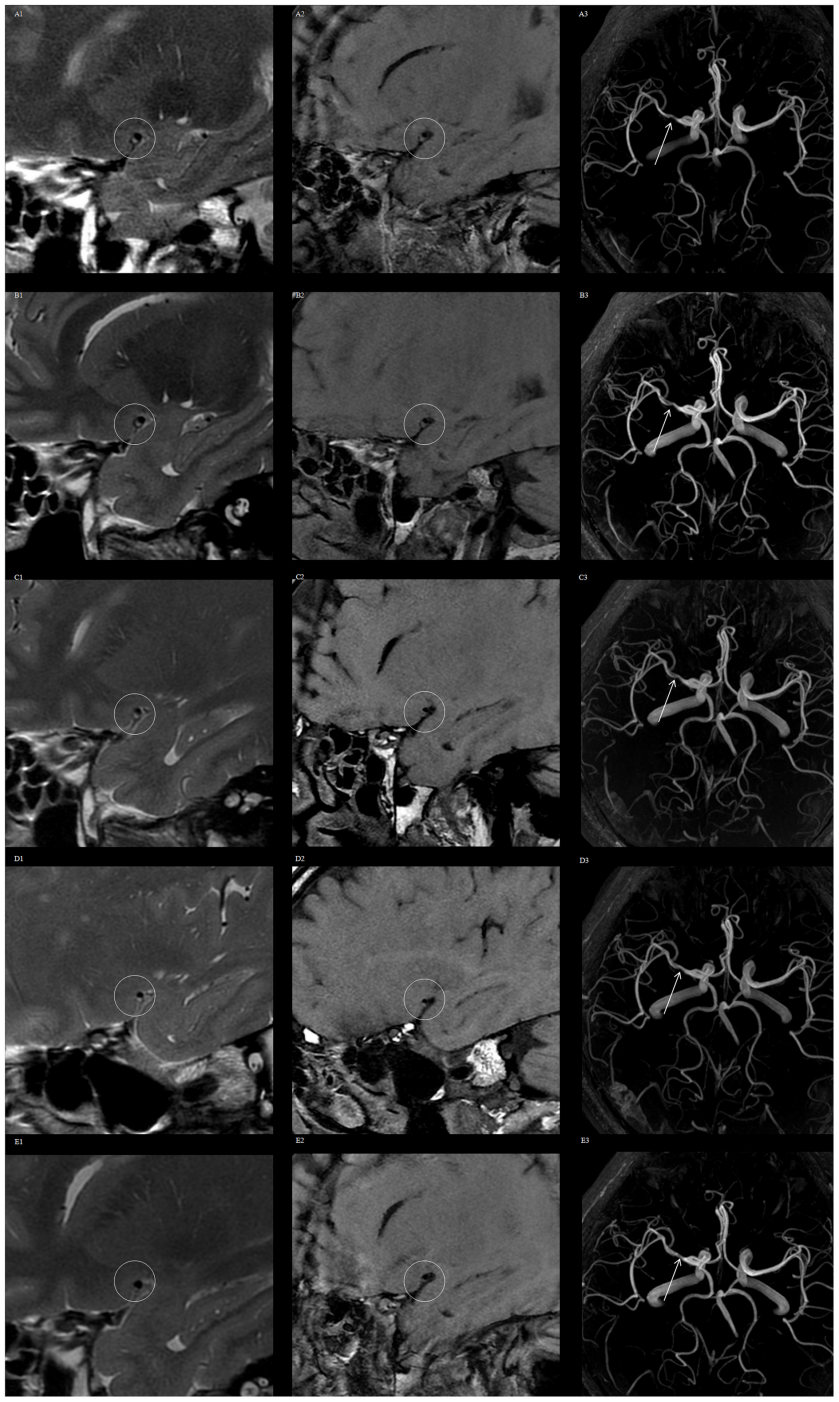
Vessel wall magnetic resonance imaging (VWMRI) of plaque changes in the right middle cerebral artery (MCA) of a patient during the follow-up period. VWMRI and MRA show no significant changes in plaque volume and luminal stenosis at baseline, 1, 3, and 6 months. The plaque volume and luminal stenosis degree were reduced at the 12-month follow-up time point. Baseline, 1-, 3-, 6-, and 12-month 3D CUBE T2 images **(A1–E1)**, 3D CUBE T1 sequence images **(A2–E2)**, and 3D TOF MRA images **(A3–E3)**.

After lipid-lowering treatment, no significant changes in VWMRI were observed at the 1- and 3-month follow-up time points compared with baseline levels; at the 6-month follow-up time point, a total of five patients (20.83%) had a reversal of MCA plaque; at the 12-month follow-up point, a total of nine patients (37.50%) had a reversal of MCA plaque (Figure 1), with a mean time to plaque reversal of 9.85 months. A total of 10 patients (41.67%) had stable MCA plaques with no significant changes during the follow-up period.

**Figure 2:**
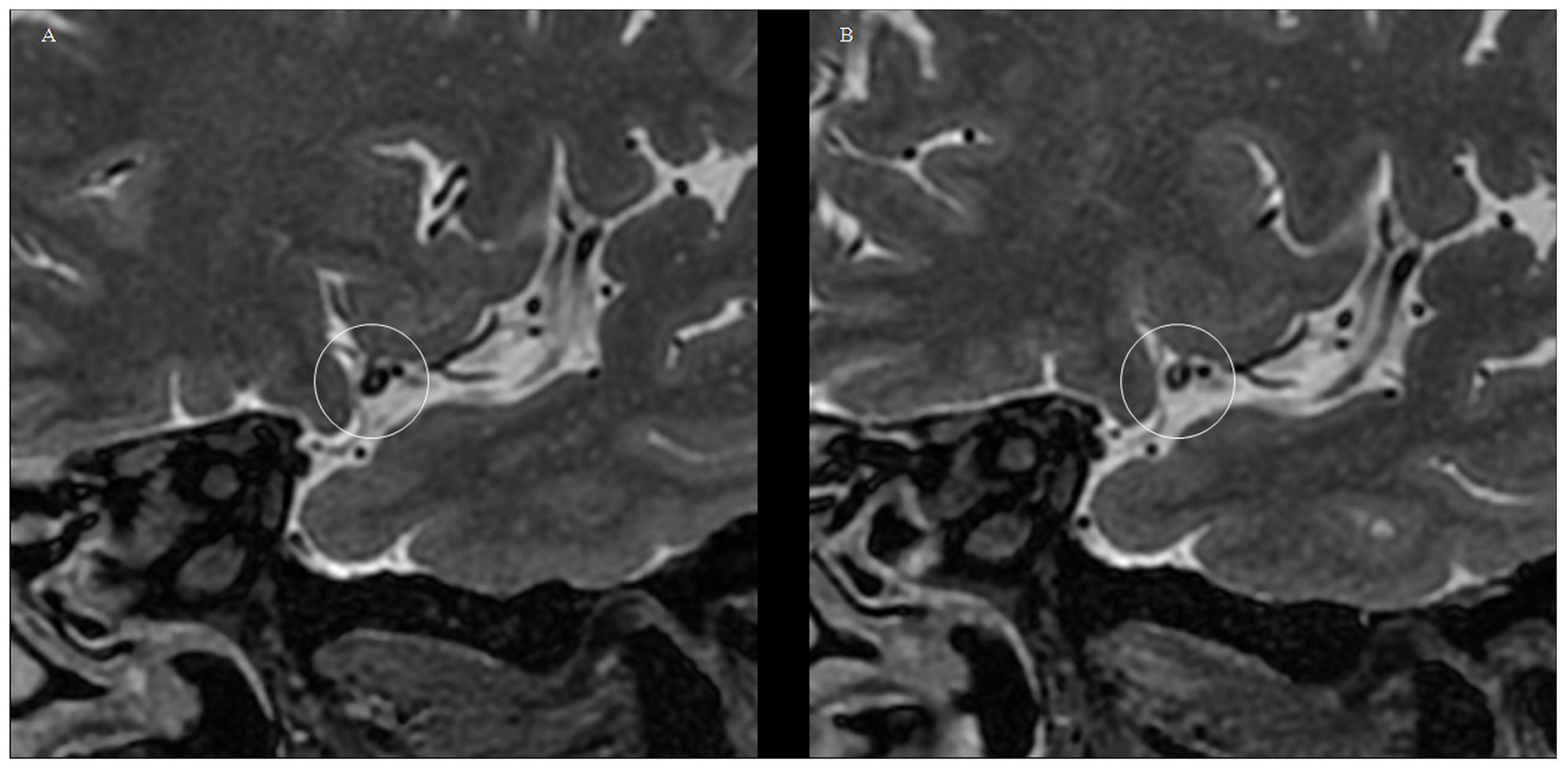
Typical VWMRI of MCA plaque changes after 12 months of atorvastatin treatment in a patient. **(A)** At baseline, the MCA plaques have a large lipid core with a thin fibrous cap and poorly displayed fibrous cap edges. **(B)** MCA plaque volume reduction at the 12-month follow-up time point. The image depicts plaque lipid core volume reduction, accompanied by fiber cap thickening, with the fiber cap edge showing more clearly.

**Figure 3:**
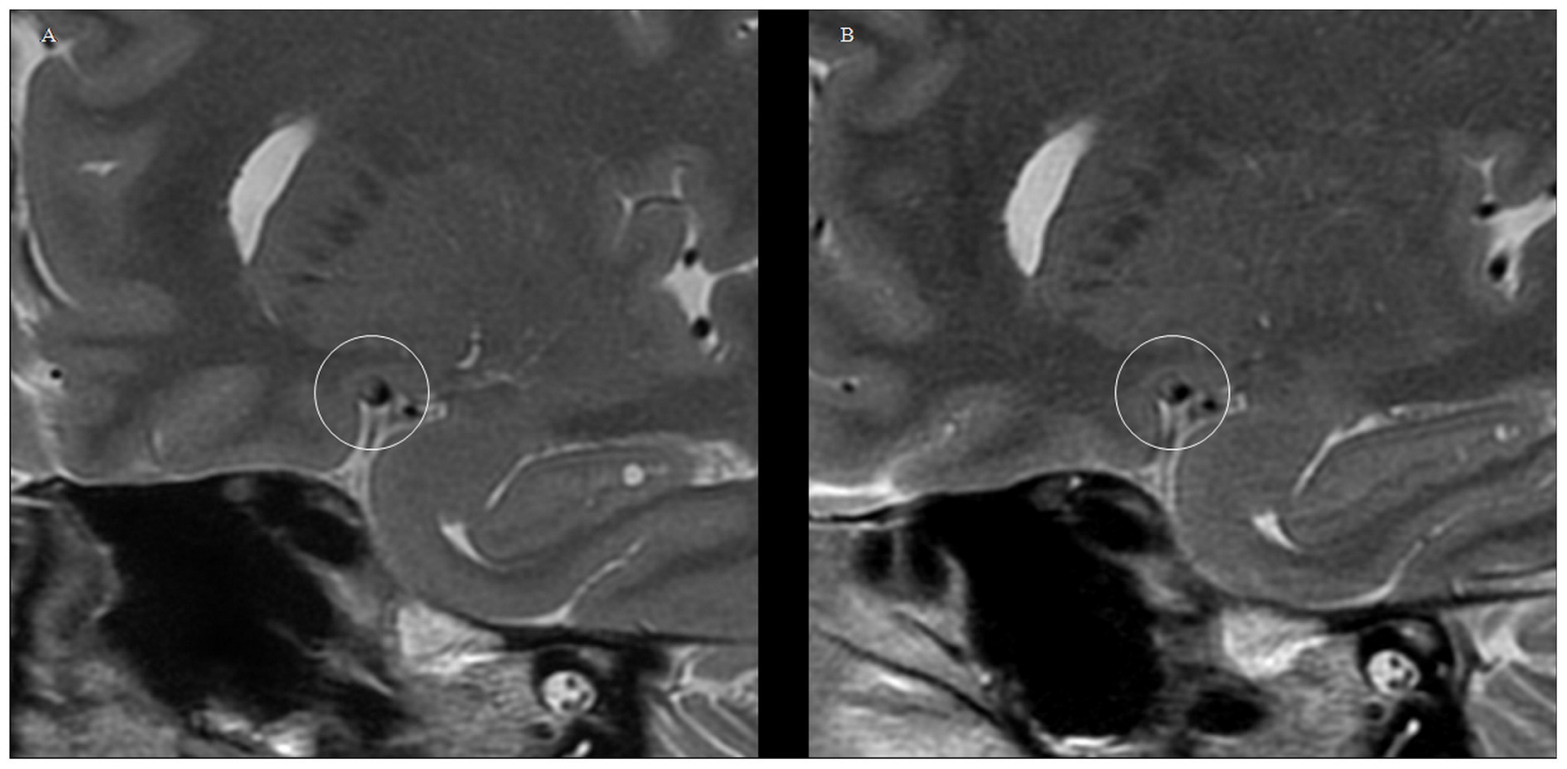
VWMRI of plaque surface changes in the MCA of a patient after receiving 12 months of atorvastatin treatment. **(A)** 2D FSE T2WI at baseline level showing the rough surface of the MCA plaque. **(B)** 2D FSE T2WI at the 12-month follow-up time point shows a smooth and well-defined MCA plaque surface.

At the follow-up endpoint, MCA stenosis (71.91 ± 15.91 vs. 66.66 ± 14.08%, *P* = 0.00) and plaque burden (80.01 ± 11.98 vs. 73.39 ± 13.46%, *P* = 0.00) were reduced in the 14 patients with plaque reversal. Although plaque reversal brought about a reduction in vessel area, it did not reverse positive vessel remodeling, and the change in RR was not significant (1.11 ± 0.04 vs. 1.08 ± 0.15, *P* = 0.29). Plaque reversal was mainly reflected in a reduction of plaque volume, which was dominated by a reduction in the internal lipid core of the plaque and an increase in fibrous cap thickness (Figure 2). Atorvastatin treatment for 12 months reversed plaques but did not change the shape and distribution of plaques. The plaque surfaces became smooth in seven patients (7/11, 63.63%) after 12 months of continuous atorvastatin treatment (Figure 3).

The clinical information of patients who experienced plaque reversal and plaque stabilization is shown in Table 4. There were no statistically significant differences in clinical outcomes between the two except for a statistically significant difference in the change in serum LDL-C concentration during the follow-up period.

**Table 4:** Clinical information of patients with different plaque properties at the follow-up endpoint.

	**Patients with plaque reversal (*N* = 14)**	**Patients with plaque stabilization (*N* = 10)**	***P***
Age (year)	57.14 ± 10.23	55.00 ± 10.83	0.81
Sex			0.28
Male	8(57.14%)	3 (30.00%)	
Female	6(42.85%)	7 (70.00%)	
Body mass index (kg/m^2^)	24.89 ± 0.89	24.86 ± 0.48	0.69
Smoking (ever)	5 (21.42%)	2 (20.00%)	0.54
Diabetes	6 (42.85%)	3 (30.00%)	0.62
Hypertension	9 (64.28%)	6 (60.00%)	0.88
Dyslipidemia	8 (57.14%)	5 (50.00%)	0.79
Alcoholism (ever)	3 (21.42)	2 (20.00%)	0.97
Final NIHSS	0^*^ (0–2)	0# (0–1)	0.37
Changes in final serum lipid concentrations (mmol/L)			
Total cholesterol	−0.71 ± 0.91	−0.70 ± 1.02	0.97
Triglycerides	−0.41 ± 0.80	−0.59 ± 0.91	0.61
LDL-C	−0.67 ± 1.02	−0.36 ± 0.28	0.03
HDL-C	0.02 ± 0.18	0.00 ± 0.41	0.82
Achieved LDL-C target (2017 AACE)	8 (57.14%)	6 (70.00%)	0.93
Achieved LDL-C target (2018 AHA)	9 (64.28%)	3(30.00%)	0.17

*NIHSS, National Institutes of Health stroke scale; LDL-C, low-density lipoprotein cholesterol; HDL-C, high-density lipoprotein cholesterol; 2017 AACE, American Association of Clinical Endocrinologists, LDL-C target <70 mg/dl, for very high-risk patients (24); 2018 AHA, American Heart Association; HDL, LDL-C target>50% reduction, for secondary atherosclerotic cardiovascular disease prevention (23)*.

* *13 patients with ischemic stroke*.

*#8 patients with ischemic stroke*.

### Correlation Analysis of Changes in VWMRI in Patients

Based on these results, we performed multiple linear regression analyses to identify independent influences on changes in plaque burden in patients treated with atorvastatin. Among the clinical and imaging variables of interest, changes in LDL-C concentration (coefficient = −0.35, SE = 0.11, *P* = 0.01) were strongly associated with changes in plaque load (Table 5).

**Table 5:** Univariate and multiple linear regression analysis of plaque burden changes in vessel wall magnetic resonance imaging (VWMRI).

	**Univariate analysis**			**Multivariable analysis**		
	**Coefficient**	***SE***	***P***	**Coefficient**	***SE***	***P***
Age (year)	−0.10	0.26	0.69			
Sex	4.61	5.38	0.40			
Malel	−2.10	5.67	0.71			
Female	−5.61	5.87	0.65			
Body mass index (kg/m^2^)	−8.87	5.30	0.11	−0.04	0.19	0.82
Smoking (ever)	−3.07	5.59	0.58			
Diabetes	−5.19	5.35	0.34			
Hypertension	−1.84	6.69	0.78			
Changes in final serum lipid concentrations (mmol/L)						
Total cholesterol	−1.59	2.95	0.59			
Triglycerides	2.28	3.42	0.51			
Low-density lipoprotein cholesterol	8.76	2.74	0.00	−0.35	0.11	0.01
High-density lipoprotein cholesterol	−1.81	9.63	0.85			
Initial stenosis rate	−0.41	0.15	0.01	0.01	0.01	0.09

*VWMRI, vessel wall magnetic resonance imaging*.

## Discussion

This study used VWMRI evaluation of plaques in patients in northern China with SMAS. It was a single-center, single-arm, prospective observational study that monitored imaging outcomes and clinical parameters in patients treated with standard doses of atorvastatin over a 12-month follow-up period. Based on the data from this study, we found that standard-dose atorvastatin consistently reduced non-HDL-C and stabilized and reversed MCA atherosclerotic plaques.

We determined that VWMRI is a valid and non-invasive method for the reliable evaluation of ICAD, which may provide additional information for clinical treatment planning.

It has been shown that disorders of lipid metabolism are major risk factors for various forms of atherosclerosis (25). Carvalho concluded that elevated serum non-HDL concentrations and reduced HDL concentrations are highly associated with the onset and progression of atherosclerosis (26). Therefore, statins have been recommended for the primary and secondary prevention of this disease as an effective form of atherosclerosis risk factor management (27). In the present study, we found that standard doses of atorvastatin consistently reduced overall serum non-HDL-C in patients. Our findings suggest that changes in LDL-C concentrations are highly correlated with plaque reversal and are an independent influence, with plaque reversal at months 6 and 12 of lipid-lowering therapy and mean LDL-C concentrations of 1.89 and 1.68 mmol/L during the same period. These concentrations are similar to the LDL-C attainment concentrations recommended by the 2017 American Association of Clinical Endocrinologists guidelines (24). Moreover, the decrease in LDL-C concentrations was higher in patients who experienced plaque reversal than in those who experienced plaque stabilization. Based on the above findings, we support the clinical use of LDL-C concentration and its alteration as key reference indicators for evaluating the effect of atorvastatin therapy and its therapeutic dose adjustment.

Our study showed that at a mean of 9.85 months of standard-dose atorvastatin treatment, 41.67% of patients had stable plaques, and 58.33% had plaque reversal, similar to the results of studies using vessel wall imaging and digital subtraction angiography as evaluation tools (11, 28). The causes of the different outcomes of plaque stabilization and reversal are complex. We analyzed common clinical features that were not statistically significantly different except for LDL-C alterations. The reasons for the different clinical outcomes may include subject-specific differences, differences in genetic diversity, and differences in associated lipid metabolism levels. The degree of lumen stenosis and plaque burden is one of the important indicators for evaluating ICAD. The greater the degree of stenosis and plaque burden, the greater the risk of ischemic stroke (29). Our study showed a mean decrease in plaque burden and lumen stenosis of 14.38 and 11.32% with 12 months of lipid-lowering treatment with atorvastatin, a change that may reduce the risk of inadequate perfusion of brain tissue distal to the responsible vessel due to lumen stenosis in patients with ICAD. According to the current evidence (30), intracranial atheromatous plaque intensification, positive remodeling, and unsmooth plaque surfaces are associated with an increased risk of ischemic stroke events, which can lead to plaque instability and increased risk of artery-to-artery embolism. Although our study confirmed that lipid-lowering therapy could reverse plaque, it could not reverse positive vascular remodeling, and there was no significant change in plaque distribution and shape. This is likely because the degree of plaque volume reduction is not sufficient to change the overall morphology of the vessel, and plaque reduction is not continuous and remains stable after a certain degree of volume reduction. In addition, positive remodeling is a compensatory mechanism made by the vessel itself, and the specific influencing factors are not yet fully clarified. In both patients with plaque reversal and those with plaque stabilization, we observed a change in plaque surface from unstable to stable and an increase in fibrous cap thickness, which would stabilize the plaque and reduce the risk of plaque rupture. This implies that even without plaque reversal, patients could benefit from long-term lipid-lowering therapy, and reduce their risk of ischemic stroke due to plaque rupture.

Several previous clinical studies using large samples have shown that the clinical benefit of intensive lipid-lowering therapy with 80 mg atorvastatin daily is significantly greater than low or moderate doses in reducing cardiovascular risk (31). However, Asians may respond more to statins than Caucasians due to genetic differences in the rate of drug metabolism (32, 33). In a recently published clinical trial, Chung et al. used 10–20 and 40–80 mg/day atorvastatin for symptomatic intracranial atherosclerotic plaques, and there were no significant differences between the two different doses in terms of stenosis, RR, and wall index (34). Therefore, we chose the atorvastatin dose of 20 mg/day for our study, and this dose is also the more commonly used treatment dose for Chinese patients with ICAD. In the evaluation of adverse effects, only one subject discontinued treatment due to failure to recover from phosphocreatine kinase elevation after discontinuation of the drug. We did not observe any other serious adverse reactions, suggesting that atorvastatin at 20 mg/day has a good safety profile and is well-tolerated for long-term use.

There are some limitations to this study: first, this is a single-center study; the patients included in this study were limited to residents of Liaoning province in northern China. It is known that genetic polymorphisms affect patients' responses to statins (35). Therefore, the generalizability of the results of this study may be limited, and the results need to be further validated in studies that include larger samples from multi-ethnic populations. Second, although this was a prospective study, the duration of follow-up in this study was only 1 year due to study funding and periodicity. Since plaque changes correlated with the duration of statin treatment, we plan to conduct a longer follow-up study of statins for SMAS to provide more study data. Third, this was a single-arm study of patients receiving standard doses of atorvastatin, and it was not possible to determine the natural course of change in SMAS plaques and the number of patients who completed the experimental design in this study was small. To verify the long-term efficacy of atorvastatin for ICAD, a multicenter, randomized, double-blind clinical trial with larger sample size and longer follow-up time is the goal of our future work. Fourth, treatment with anti-platelet, anti-hypertensive, and glucose drugs in addition to atorvastatin may interfere with the progression of atherosclerotic plaques in SMAS, and because of the small number of patients in this study, this issue needs to be further addressed in studies that include larger numbers of patients. Fifth, only the T1/T2 sequence was selected for the study. The T1-enhanced sequence may provide a more accurate display of the vascular lumen and plaques (36). However, injection of contrast medium is invasive, and there is a risk of gadolinium deposition in the brain (37), and the vast majority of patients were not willing to undergo enhanced MRI scans. Finally, although all patients in this study received anti-platelet therapy, we did not perform platelet agglutination tests to assess the effect of anti-platelet drug therapy. All patients were monitored for plasma prothrombin time, plasma fibrinogen, activated partial thromboplastin time, and plasma prothrombin time and did not show any abnormalities during the follow-up period.

In conclusion, VWMRI can accurately characterize changes in MCA plaques after lipid-lowering therapy and provide a reliable basis for the clinical diagnosis and treatment of ICAD. In patients with SMAS in northern China, long-term regular treatment with standard doses (20 mg/day) of atorvastatin was effective in stabilizing and reversing plaque, while changes in LDL-C were an independent factor affecting plaque stabilization and retraction.

## Data Availability Statement

The raw data supporting the conclusions of this article will be made available by the authors, without undue reservation.

## Ethics Statement

The studies involving human participants were reviewed and approved by Ethics Committee of the Fourth Hospital of China Medical University (EC-2019-KS-016). The patients/participants provided their written informed consent to participate in this study.

## Author Contributions

YWu: guarantor of integrity of the entire study, study concepts and design, literature research, clinical studies, experimental studies, data analysis, statistical analysis, manuscript preparation, and manuscript editing. FL: clinical studies, experimental studies, data analysis, and statistical analysis. YWa: clinical studies, experimental studies, and data analysis. TH: experimental studies and data analysis. HG: manuscript preparation and manuscript editing. All authors contributed to the article and approved the submitted version.

## Funding

The project was funded by Natural Science Foundation of Liaoning Province (2019-ZD-0762).

## Conflict of Interest

The authors declare that the research was conducted in the absence of any commercial or financial relationships that could be construed as a potential conflict of interest.

## Publisher's Note

All claims expressed in this article are solely those of the authors and do not necessarily represent those of their affiliated organizations, or those of the publisher, the editors and the reviewers. Any product that may be evaluated in this article, or claim that may be made by its manufacturer, is not guaranteed or endorsed by the publisher.
